# Combination of the CRAC Channel Inhibitor CM4620 and Galactose as a Potential Therapy for Acute Pancreatitis

**DOI:** 10.1093/function/zqae017

**Published:** 2024-04-05

**Authors:** Siân Lewis, David L Evans, Tetyana T Tsugorka, Shuang Peng, Ken Stauderman, Oleg Gerasimenko, Julia Gerasimenko

**Affiliations:** Cardiff School of Biosciences, Cardiff University, Museum Avenue, Cardiff CF10 3AX, UK; Cardiff School of Biosciences, Cardiff University, Museum Avenue, Cardiff CF10 3AX, UK; Cardiff School of Biosciences, Cardiff University, Museum Avenue, Cardiff CF10 3AX, UK; School of Sport and Health Sciences, Guangzhou Sport University, Guangzhou 510500, China; CalciMedica, Inc., La Jolla, CA, 92037, USA; Cardiff School of Biosciences, Cardiff University, Museum Avenue, Cardiff CF10 3AX, UK; Cardiff School of Biosciences, Cardiff University, Museum Avenue, Cardiff CF10 3AX, UK

**Keywords:** Acute pancreatitis, Orai1/CRAC, galactose, combination, therapy

## Abstract

Acute pancreatitis (AP) is a life-threatening inflammatory disease with no specific therapy. Excessive cytoplasmic Ca^2+^ elevation and intracellular ATP depletion are responsible for the initiation of AP. Inhibition of Ca^2+^ release–activated Ca^2+^ (CRAC) channels has been proposed as a potential treatment, and currently, a novel selective CRAC channel inhibitor CM4620 (Auxora, CalciMedica) is in Phase 2b human trials. While CM4620 is on track to become the first effective treatment for AP, it does not produce complete protection in animal models. Recently, an alternative approach has suggested reducing ATP depletion with a natural carbohydrate galactose. Here, we have investigated the possibility of using the smallest effective concentration of CM4620 in combination with galactose. Protective effects of CM4620, in the range of 1-100 n m, have been studied against necrosis induced by bile acids, palmitoleic acid, or l-asparaginase. CM4620 markedly protected against necrosis induced by bile acids or asparaginase starting from 50 n m and palmitoleic acid starting from 1 n m. Combining CM4620 and galactose (1 m m) significantly reduced the extent of necrosis to near-control levels. In the palmitoleic acid-alcohol–induced experimental mouse model of AP, CM4620 at a concentration of 0.1 mg/kg alone significantly reduced edema, necrosis, inflammation, and the total histopathological score. A combination of 0.1 mg/kg CM4620 with galactose (100 m m) significantly reduced further necrosis, inflammation, and histopathological score. Our data show that CM4620 can be used at much lower concentrations than reported previously, reducing potential side effects. The novel combination of CM4620 with galactose synergistically targets complementary pathological mechanisms of AP.

## Graphical Abstract

**Graphical Abstract:**
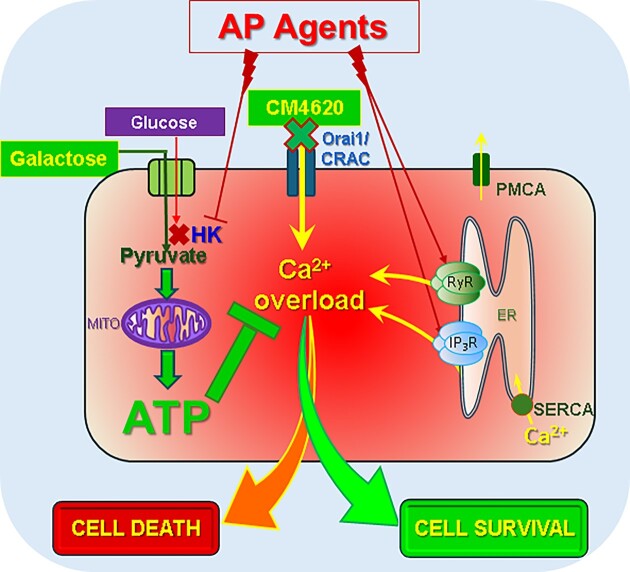

## Introduction

Acute pancreatitis (AP) is a life-threatening, inflammatory human disease with incidence rates of up to 100 people per 100,000 per annum and increasing numbers of pediatric cases.^1^,^2^ The general mortality rate is typically up to 5%; however, advanced forms of AP develop in approximately 20% of patients with prolonged hospitalization and more severe complications characterized by significant pancreatic necrosis, a systemic inflammatory response, multiple-organ failure, and an increased mortality of 30%.^1^,^3^^-^ ^6^ Without a specific therapeutic available in clinics, this devastating disease represents an increasing burden for society and healthcare services.

Gallstone biliary disease and excessive alcohol consumption are the leading causative factors of AP, responsible for approximately 70%-80% of cases.^7^^-^ ^10^ Gallstone obstruction of the bile duct can result in bile reflux into the pancreatic duct or an increase in pressure, exposing the pancreas to biliary components that induce pancreatic acinar cell (PAC) injury.^11^^-^ ^13^ Other known causes of AP are either hereditary^13^ or due to side effects of some drugs, such as cancer drugs based on l-asparaginase (ASNase), an essential treatment received by patients suffering from acute lymphoblastic leukemia (ALL), the most common type of cancer affecting children.^14^ The development of ASNase-induced AP (AAP) in up to 10% of cases becomes the most common reason for ending this life-saving treatment.^15^^-^ ^20^

It has been established previously that AP-eliciting agents, including bile acids, alcohol metabolites, and ASNase, cause cytosolic Ca^2+^ overload in PACs as a result of excessive Ca^2+^ release from the internal stores followed by Ca^2+^ entry mainly through Orai1/Ca^2+^ release–activated Ca^2+^ (CRAC) channels.^21^ The aberrant Ca^2+^ signaling leads to premature intracellular activation of digestive proenzymes (as opposed to normal activation occurring when they are secreted into the pancreatic acinar lumen) and loss of cellular ATP due to mitochondrial dysfunction.^22^^-^ ^25^ This results in PAC necrosis and tissue inflammation.^26^

In recent years, various human diseases have been associated with abnormal CRAC channel activity, including severe disorders of the immune system, allergies, myocardial infarction, thrombosis, Alzheimer’s disease, cancer, and AP.^27^^-^ ^33^ The recognition of store-operated Ca^2+^ entry (SOCE) as a potential therapeutic target for AP dates back to as early as 2000.^34^ The pharmacological development of specific CRAC channel inhibitors for AP treatment has significantly expanded over recent years.^35^ The substantial therapeutic appeal of targeting CRAC channels is due to the dependence of intracellular protease activation on cytosolic Ca^2+^ overload, which occurs after abnormal Ca^2+^ depletion of the endoplasmic reticulum (ER) and excessive Orai1/CRAC channel-mediated Ca^2+^ entry.^21^,^29^,^36^^-^ ^38^

CM4620 (zegocractin), a novel small molecule Orai1/CRAC channel inhibitor developed by CalciMedica, has completed a Phase 2 clinical trial for treating moderate to severe AP and is the most advanced pharmaceutical in clinical development for the treatment of AP.^39^,^40^ The effectiveness of CM4620 at inhibiting SOCE in PACs, immune cells, and pancreatic stellate cells (PSCs) of a mouse, rat, and human origin, as well as in *in vivo* mouse and rat models of cerulein-induced AP, was recently demonstrated.^42^ Intravenous infusion of Auxora, the intravenous (IV) emulsion formulation of CM4620, in an *in vivo* rat model of pancreatitis significantly diminished pancreatic edema, acinar cell vacuolization, intrapancreatic trypsin activity, and acinar cell necrosis. The expression of inflammatory cytokines in pancreas and lung tissues and cytokine generation in human peripheral blood mononuclear cells and rodent PSCs were markedly decreased, thus revealing a role for Orai1/STIM1 in the cellular inflammatory pathways involved in AP. However, the efficacy of CM4620 on pancreatic histopathology was not 100%, and higher doses or long-term application of this compound is potentially problematic due to the inhibitory effects of CM4620 on immune and other cells that could lead to unwanted immunological, muscular, or intestinal consequences.^32^,^41^,^42^

In *in vitro* experiments performed by Waldron et al.,^42^ the reduction of cerulein-induced Ca^2+^ entry in mouse PACs in the presence of 1 μm CM6420 was up to 70%, leaving room for a potential reduction of an effective CM4620 concentration to lower levels. However, reduced inhibition of calcium entry could also affect the effectiveness of the drug treatment.

To enhance the treatment efficacy of reduced concentrations of CM4620, it might be possible to use it in combination with other proposed treatments. Recently, we have shown that energy supplements such as galactose and pyruvate can provide a high degree of protection against pancreatic necrosis in PACs by restoring ATP production,^25^ as discussed in detail in several reviews.^1^,^5^,^43^ ATP metabolism plays a major role in Ca^2+^ homeostasis and regulation of PAC function; therefore, maintaining cytoplasmic ATP levels is an ultimate condition of cell survival.^1^,^5^ Due to cytosolic and mitochondrial Ca^2+^ overload during the initial stages of AP, the ATP production by mitochondria is seriously affected causing ATP depletion at a cellular level. For the first time, we have provided detailed evidence of the role of glycolysis in AP *in vitro* and *in vivo*.^24^,^25^ We have demonstrated that a potential mechanism involves the inhibition of hexokinases (HKs), the enzymes that convert glucose into glucose-6-phosphate, by several well-known AP-inducing agents, namely bile acids, alcohol, and asparaginase.^25^ Addition of pyruvate or galactose as a source of energy that acts independently of HKs significantly reduced sustained Ca^2+^ elevations, ATP loss, and PAC necrosis induced by alcohol metabolites, bile acids, or ASNase.^25^ Galactose markedly reduced all main histological parameters of the damage to pancreatic tissue in experimentally induced fatty acid ethyl ester (FAEE)-AP^1^,^33^ and AAP^25^  *in vivo* models of AP. The safety of galactose administration in humans, even at high m m concentrations, has also been shown.^48^ At relatively high concentrations (ie, up to 70 m m), galactose is present in a variety of lactose-free dairy products^44^ and is regularly consumed by large proportions of the population. Free galactose is also a component of breast milk at m m concentrations as well as existing in formula milk at concentrations of 2-4 m m.^45^,^46^ Galactose has been used in a number of clinical trials^47^,^59^ with a maximum dose of 1.5 g/kg for up to 18 wk.^48^,^57^,^59^,^60^

Therefore, a combination of such treatments that target very different mechanisms would be highly appropriate and have a high chance of success.

We have aimed our study at investigating the possibility of finding the lowest effective concentration of CM4620 and testing whether its protective effects in AP could be enhanced by using a combination of CM4620 and galactose. Such a combination could provide effective protection against pathological effects elicited by AP-inducing agents and at the same time minimize potential adverse effects of CM4620.

## Materials and Methods

### Materials

l-Asparaginase was purchased from Abcam, Cambridge, UK. CM4620 was a gift from CalciMedica, La Jolla, California. Cyclopiazonic acid (CPA) was obtained from Tocris, Bristol, UK. Fluo-4 AM and propidium iodide (PI) were purchased from Thermo Fisher Scientific, Paisley, UK. All other reagents were from Sigma-Aldrich, UK.

### Experimental Mouse Model of AP

All regulated procedures involving animals were performed in compliance with the UK Home Office regulations under the Animal (Scientific Procedures) Act, 1986. C57BL6/J male mice (6-8 wk old, 23 ± 3 g) were obtained from Charles River Laboratories (UK). They were housed with corn cob bedding and an enriched environment, which included nesting material and cardboard tunnels. Mice were randomly and blindly allocated per experimental group from the available stock.

For the induction of experimental alcohol-induced AP, the mice received 2-hourly intraperitoneal (IP) injections of palmitoleic acid (POA; 150 mg/kg) combined with ethanol (1.35 g/kg) to induce AP (FAEE-AP) (positive control).^25^,^33^ In order to reduce potential damage to peritoneal organs at the injection site, 200 μL sterile phosphate-buffered saline (PBS) was injected immediately before the ethanol/POA injection. Control mice (negative control) received 2-hourly IP injections of PBS alone. Twenty-four hours prior to FAEE-AP induction, analgesia was given for compassionate reasons by oral administration of 2.5 μg/mL buprenorphine hydrochloride. In the CM4620 treatment group, mice were co-administered IP injections of 0.1 mg/kg CM4620 (dissolved in a mixture of dimethylsulfoxide (DMSO) and PBS) together with the first ethanol/POA injections, which were given 2 times at 1-h intervals.^25^,^33^ In the galactose CM4620 treatment group, the drinking water was supplemented with 100 m m galactose 24 h before and during co-administration of IP injections of 0.1 mg/kg CM4620 together with the first ethanol/POA injections, which were given 2 times at 1-h intervals. Animals were sacrificed 24 h after the first injection and pancreatic tissue was extracted for histological analysis, to assess the severity of FAEE-AP. Treatment groups consisted of ≥4 mice/group.

Pancreatic tissues were fixed in 4% formaldehyde, 24 h before processing. Fixed pancreatic tissues were then embedded in paraffin and stained with hematoxylin and eosin (H&E). A total of 15 or more random fields (magnification, x200) per slide were assessed for edema, acinar cell necrosis, and inflammatory cell infiltration by 2 independent investigators in a blinded manner using a 0-3 grading scale, as previously described.^49^

### Isolation of PACs

PAC isolation was performed as described previously.^50^ Briefly, the pancreas was rapidly dissected from a mouse and washed twice in standard 4-(2-Hydroxyethyl)piperazine-1-ethanesulfonic acid sodium salt (NaHEPES) buffer solution (140 m m NaCl; 4.7 m m KCl; 10 m m HEPES; 1 m m MgCl_2_; 10 m m d(+)glucose, 1 m m CaCl_2_, pH 7.2). The pancreas was injected with collagenase solution and incubated for 5-6 min at 37°C. After incubation, the tissue was manually agitated by pipetting in NaHEPES buffer. PACs were collected and centrifuged at 200 x *g* for 1 min. The supernatant was discarded, and the cell pellet was resuspended in fresh NaHEPES buffer solution and centrifuged a second time at 200 x *g* for 1 min. The final cell pellet was suspended in fresh NaHEPES buffer and used for experiments. All experiments were conducted at room temperature (22°C).

### Cytosolic Ca^2+^ Measurements

Freshly isolated PACs were loaded for 45 min with the Ca^2+^-sensitive fluorescent probe Fluo-4 AM (5 µm). The cells were adhered to glass coverslips and continuously perfused, in a flow chamber, with an NaHEPES-based extracellular solution.^50^ Fluorescence was imaged over time using a Leica SP5 2-photon or Leica TCS SPE confocal microscopes (Leica Microsystems, Milton Keynes, UK; 40x oil objective; excitation, 488 nm; emission, 510-560 nm). A Scientifica imaging system based on an inverted Olympus IX71 system (Tokyo, Japan; 40x oil objective; excitation 470 nm; emission 515-560 nm; WinFluo software was used for data recording) was also used.

### Necrosis Measurements

Propidium iodide (PI) was used to visualize and count cell necrosis levels with the help of a Lecia confocal microscope TCS SPE. Positive PI staining (excitation 532 nm, emission: 585-705 nm), represented by intense red nucleus staining due to plasma membrane rupture, allowed for the detection of necrotic cells. A total of 20-25 images, per condition, were taken and the total number of cells was calculated by counting the number of necrotic (PI positive staining) and viable (PI negative staining) cells. At least 3 independent experiments (*N *= 3) for each condition were performed (>100 cells per condition). This enabled the average percentage of necrotic cells of the total number of cells ± SEM to be calculated and presented as a bar chart.^21^

### Statistical Analysis

Outcome measures (mean ± SEM) were analyzed using the Pearson test to determine the normality of data distribution. Statistical significance and *P*-values were calculated using a one-way ANOVA or Kruskal-Wallis tests, with the significance threshold set at .05 and asterisks representing the range (*P < .05, **P < .01, ***P < .001, ^****^P < .0001).

## Results

### Low Submicromolar Concentrations of CM4620 Provide Significant Inhibition of Ca^2+^ Entry in PACs

**Figure 1.**
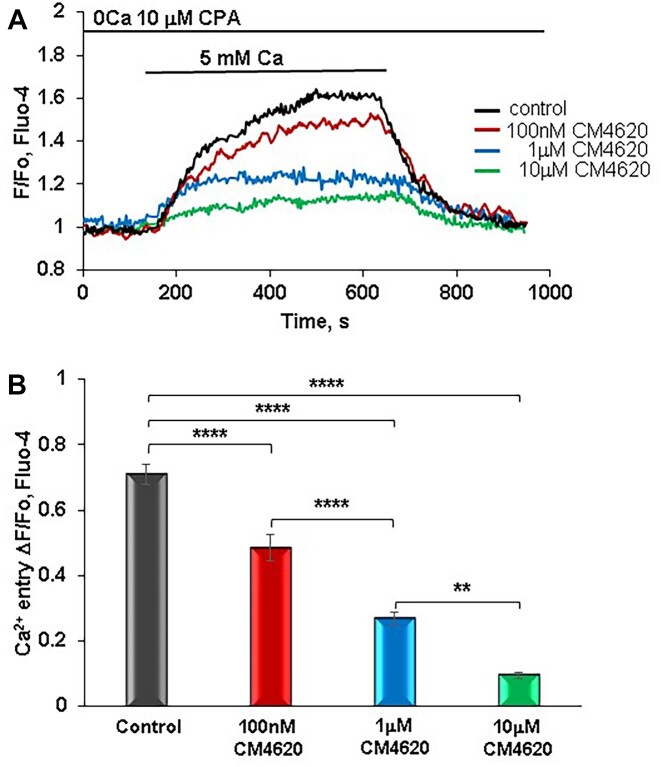
Submicromolar concentrations of CM4620 significantly inhibit Ca^2+^ entry in PACs in a dose-dependent manner. (A) Representative traces depict Ca^2+^ entry in PACs in the presence or absence of treatment with different concentrations of CM4620 (100 n m, 1 µm, or 10 µm). To observe Ca^2+^ entry, cells were treated with CPA (10 μm) in nominally Ca^2+^-free standard buffer to deplete the ER Ca^2+^ followed by the addition of 5 m m Ca^2+^. (B) Effect of CM4620 on cytosolic Ca^2+^ ([Ca^2+^]_i_) amplitude change (Δ*F*/*F*_o_) as a result of Ca^2+^ entry in PACs. CM4620 significantly reduced the average amplitudes of Ca^2+^ signals representing the extent of Ca^2+^ entry in PACs at 100 n m (red bar, *n* = 24), 1 μm (blue bar, *n* = 23), or 10 μm (green bar, *n* = 16) as compared to control cells (untreated with CM4620, dark gray trace, *n* = 39) (*P* < .0001). Cells were loaded with Fluo-4 AM. Bars presented as mean ± SEM.

To investigate a potential protective effect of concentrations of CM4620 lower than reported before against excessive Ca^2+^ entry in AP, we have compared Ca^2+^ influx levels in freshly isolated control (untreated) PACs and cells pretreated with 100 n m, 1 µm, or 10 μm CM4620 (Figure 1A). Freshly isolated PACs loaded with Fluo-4 AM were initially perfused with standard buffer in the absence of external Ca^2+^ and then perfused in the presence of the specific Sarcoendoplasmic Reticulum Calcium ATPase (SERCA) pump inhibitor CPA (10 µm) to deplete ER Ca^2+^ stores. Thereafter, to observe Ca^2+^ entry, 5 m m CaCl_2_ was added, resulting in a considerable rise of [Ca^2+^]_i_ representing Ca^2+^ influx (Figure 1A). After 500 s when a stable [Ca^2+^]_i_ plateau was reached, cells were perfused with a solution of nominally free Ca^2+^, causing [Ca^2+^]_i_ recovery to the baseline. In other experiments, cells were preincubated for 30 min with different concentrations of CM4620 prior to administration of the solution with 5 m m Ca^2+^. CM4620 at 1 or 10 μm CM4620 significantly inhibited the amplitude of Ca^2+^ entry as compared to untreated control cells (*P* < .0001, Figure 1A and B). These data are in line with previously published results.^33^,^42^ However, we have found that the much lower concentration of 100 n m CM4620 was also able to cause a significant reduction of Ca^2+^ influx (*P* < .0001, Figure 1A and B), suggesting that it might be possible to reduce effective concentrations of CM4620 without a substantial loss of its ability to suppress Ca^2+^ entry.

### Effects of Low Concentrations of CM4620 on Physiological and Supramaximal Ca^2+^ Responses Elicited by ACh in PACs

**Figure 2.**
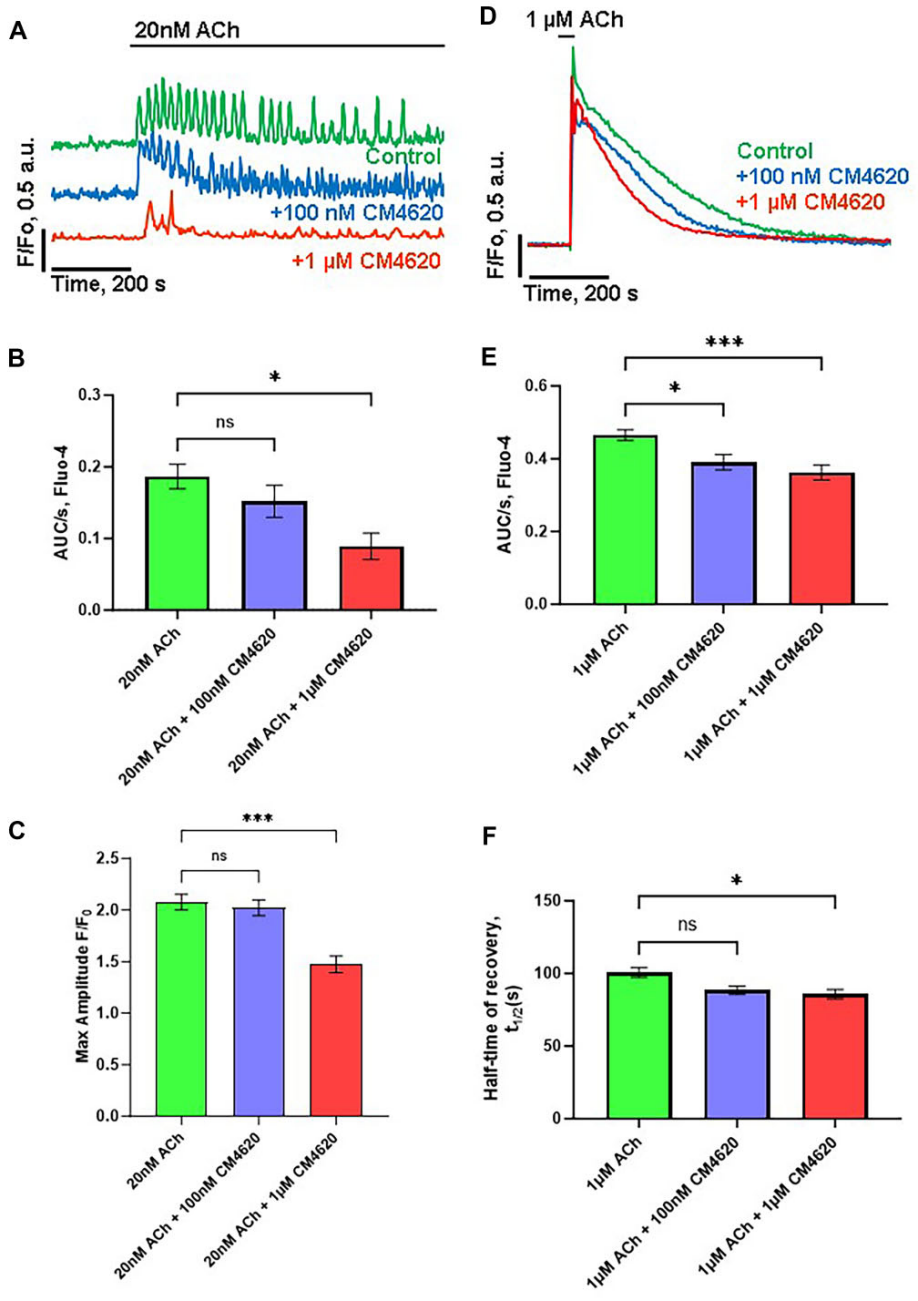
CRAC channel inhibitor CM4620 at a concentration of 1 μm significantly reduces cytosolic Ca^2+^ signals induced by physiologically relevant concentrations of ACh. (A) Representative traces depict [Ca^2+^]_i_ oscillations induced by 20 n m ACh in control cells (green trace, *n* = 33), cells preincubated with 100 n m CM4620 (blue trace, *n* = 21), or in cells preincubated with 1 μm CM4620 (red trace, *n* = 9). (B) Quantitative analysis of integrated [Ca^2+^]_i_ signals shown in (A) by calculation of averaged areas under the curve of ACh-elicited [Ca^2+^]_i_ responses (time interval 800 s from the addition of ACh) in the presence of 100 n m CM4620 (blue bar) as compared to control (*P* > .05) or in the presence of 1 μm CM4620 (red bar, *P < .05) as compared to control (green bar). (C) Comparison of the maximal amplitudes of the oscillations shown in (A). Averaged maximal amplitudes of ACh-induced [Ca^2+^]_i_ signals in cells preincubated with 100 n m CM4620 (blue bar) compared to control (*P* > .05) or in the presence of 1 μm CM4620 (red bar, ***P < .0001) as compared to control (green bar). (D) Representative traces demonstrate [Ca^2+^]_i_ global signals evoked by 1 µm ACh in control cells (green trace, *n* = 60), cells preincubated with 100 n m CM4620 (blue trace, *n* = 19), or cells preincubated with 1 µm CM4620 (red trace, *n* = 27). (E) Comparison of the average areas under the curve of [Ca^2+^]_i_ changes induced by a high concentration of ACh (1 μm) shown in (D) in the presence of 100 n m CM4620 (blue bar) or 1 µm ACh (red bar). The responses to ACh in the presence of 100 n m CM4620 were significantly lower (*P < .05) than in control and highly significantly lower in the presence of 1 µm CM4620 (***P < .001). (F) Comparison of the half-time of [Ca^2+^]_i_ recovery following maximal stimulation with 1 μm ACh shown in (D) in the presence of either 100 n m CM4620 (*P *> .05, blue bar) or 1 µm ACh (*P < .05, red bar). Data represent mean ± SEM. Cells were loaded with Fluo-4 AM. Experiments were performed in a standard buffer containing 1 m m CaCl_2_.

The secretagogue acetylcholine (ACh) plays an important role as a physiological stimulus that controls Ca^2+^ signaling in PACs. Therefore, we have investigated a possible effect of 100 n m and 1 µm (Figure 2A-F) of CM4620 on cytosolic Ca^2+^ signals evoked by a low physiologically relevant concentration of ACh. In control experiments, 20 n m ACh evoked transient cytosolic Ca^2+^ oscillations in PACs loaded with Fluo-4 AM (Figure 2A, green trace). Stimulation with a supramaximal secretagogue concentration (ACh 1 μm) evoked a global cytosolic Ca^2+^ response (Figure 2D, green trace). Both types of responses were similar to those published previously.^50^ Following pretreatment of cells with CM4620 for 30 min, the repetitive, local [Ca^2+^]_i_ spikes produced by 20 n m ACh were not inhibited by 100 n m CM4620 (*P *> .05) but markedly reduced by 1 μm CM4620 (*P *< .05) when quantified by calculation of “area under the curve” (Figure 2B). The averaged maximal amplitudes of cytosolic Ca^2+^ signals elicited by 20 n m ACh were not significantly lower (*P *> .05) in cells pretreated with 100 n m CM4620 but significantly reduced by 1 μm CM4620 (*P *< .05) as compared to control cells (Figure 2C). The degree of reduction could be due to partial depletion of the ER during incubation (30 min) with CM4620, suggested by the results shown in Figure 2D and E. A marked reduction was observed in average areas under the curve of Ca^2+^ signals in PACs stimulated with supramaximal concentrations of ACh (1 μm) (Figure 2D and E), in the presence of 100 n m (*P *< .05) or 1 μm CM4620 (*P *< .001) as compared to control cells. Interestingly, the rate of recovery of Ca^2+^ signals in cells preincubated with 1 µm CM4620 to baseline levels after response to 1 μm ACh was significantly faster (97.7 s ± 6.68, *P* < .05) than in untreated control cells (141.6 s ± 7.67) (Figure 2F). However, in cells treated with 100 n m CM4620, the rate of Ca^2+^ recovery was not significantly different (*P *> .05) from control cells (Figure 2F).

### Effects of Submicromolar Concentrations of CM4620 in Combination with Galactose on Necrosis in PACs

**Figure 3.**
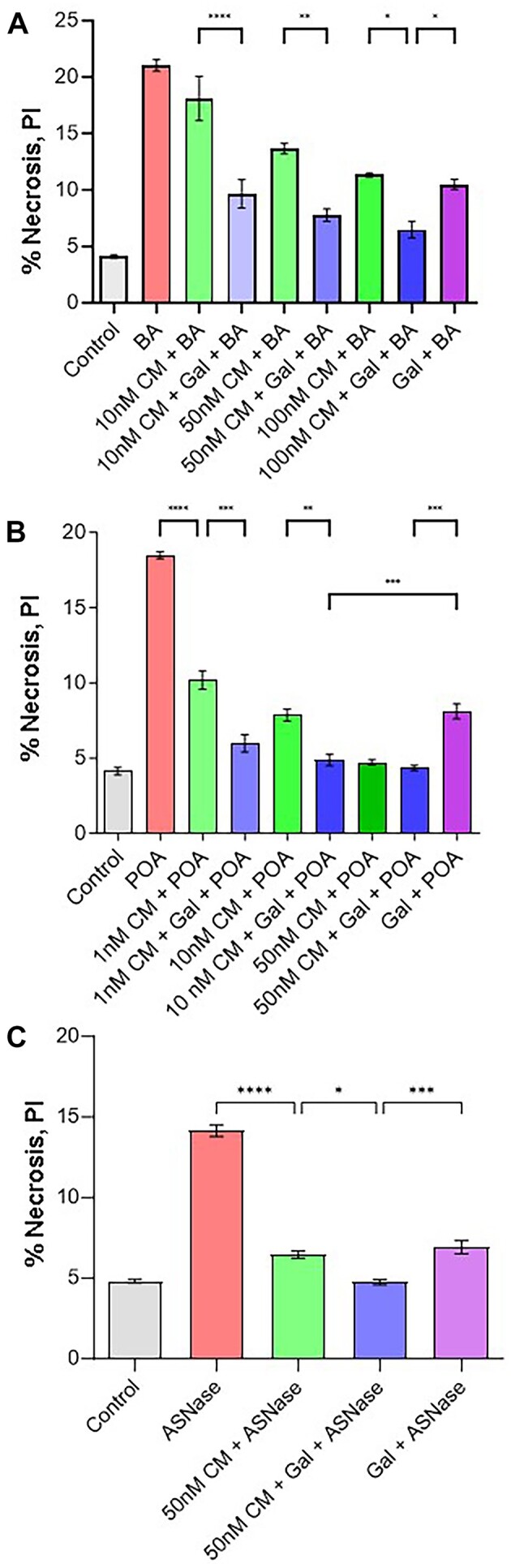
A combination of galactose and reduced concentrations of CM4620 provides significant protection against necrosis in PACs induced by sodium choleate, POA, or ASNase. (A) The treatment of cells with 10 n m CM4620 did not protect against sodium choleate (BA) (0.06%)–evoked necrosis as compared to the necrosis level produced by BA alone (*P* > .05). However, a combination of 10 n m CM4620 and 1 m m galactose does markedly diminish cell death induced by BA as compared to a combination of 10 n m CM4620 and BA (*P* < .0001). CM4620 at concentrations of 50 and 100 n m significantly reduced PAC necrosis induced by BA (*P* < .0001). Galactose (1 m m) supplement has also significantly reduced BA-elicited necrosis further to nearly the control level in cells treated with 50 or 100 n m CM4620 (*P* < .05 and *P *> .05, respectively, when compared to control). The combination of galactose with CM4620 (50 and 100 n m) has significantly increased the protection provided by CM4620 alone (*P *< .01 and *P* < .05, respectively). Treatment of cells with 1 m m galactose alone very highly significantly decreased necrosis induced by BA (*P* < .0001). At least 3 experiments per group were performed with more than 150 cells in each sample. Data presented as mean ± SEM. (B) POA (30 μm)–evoked necrosis is significantly reduced by treatment of cells with CM4620 in a dose-dependent manner (1, 10, and 50 n m) (*P* < .0001). The addition of 1 m m galactose to cells treated with 1 and 10 n m CM4620 was effective in significantly reducing POA-induced necrosis in cells, as compared to CM4620 treatments alone (*P* < .001 and *P* < .01, respectively). Also, 50 n m CM4620 was able to reduce necrosis to the control level (*P* > .05). The combination of galactose (1 m m) with CM4620 (1 and 10 n m) significantly increased the protection provided by CM4620 alone (*P *< .001 and *P* < .01, respectively). No further significant reduction was found between 50 n m CM4620 alone and in combination with 1 m m galactose (*P* > .05). Treatment of cells with 1 m m galactose alone very highly significantly decreased necrosis induced by POA (*P* < .0001). At least 3 experiments per group were performed with more than 150 cells in each sample. Data presented as mean ± SEM. (C) CM4620 (50 n m) significantly reduced the extent of necrosis (*P* < .0001) induced by ASNase (200 U/mL). Using a combinational approach, applying a mixture of galactose (1 m m) and 50 n m CM4620 reduced ASNase-evoked necrosis further (*P* < .05 as compared to 50 n m CM4620 alone). A combination of CM4620 and galactose reduced ASNase-induced necrosis to the control level (*P* > .05). Galactose (1 m m) alone was able to significantly reduce ASNase-elicited cell necrosis (*P* < 0001). At least 3 experiments per group were performed with more than 150 cells in each sample. Data presented as mean ± SEM.

The ability of micromolar concentrations of CM4620 to significantly reduce mouse PAC necrosis elicited by AP-inducing agents was demonstrated previously.^33^,^42^ In the present study, we extended previous results by testing the effectiveness of nanomolar concentrations of CM4620 alone or in combination with 1 m m galactose to reduce PAC necrosis (Figure 3). Concentrations of CM4620 (1-100 n m) have been tested under the pathological conditions induced by the nonoxidative ethanol metabolite POA or a mixture of bile acids (sodium choleate, BA) or ASNase.^25^ In our experiments, the level of PAC necrosis elicited by BA (0.06%) was very significantly reduced by either 50 or 100 n m CM4620 (*P* < .0001) (Figure 3A). Treatment with 10 n m CM4620 did not significantly affect the BA-induced levels of necrosis (*P *> .05) (Figure 3A). However, the mixture of 10 n m CM4620 and 1 m m galactose provided highly significant protection against BA-elicited necrotic cell death (*P* < .0001) as compared to cell death levels in the presence of 10 n m CM4620 alone (Figure 3A). There was no significant difference between BA and galactose and the combination of BA, galactose, and 10 n m CM4620 (*P *> .05), in line with the first observation of insignificant inhibition by 10 n m CM4620 alone (Figure 3A). Combining galactose with either 50 or 100 n m CM4620 very effectively inhibits BA-induced necrosis (*P* < .01 and *P* < .05, respectively) (Figure 3A). In line with our previous data,^25^ 1 m m galactose very significantly inhibited necrosis induced by BA alone (*P* < .0001).

We have also tested the protective effect of CM4620 against POA-induced necrosis (Figure 3B). In comparison to the average necrosis level of untreated control cells (gray column), treatment with 30 µm POA substantially increased the number of necrotic cells (red column) (*P* < .0001) (Figure 3B). Pretreatment of PACs with 1, 10, or 50 n m CM4620 (green columns) significantly reduced levels of necrosis compared to POA alone (*P* < .0001). Furthermore, 50 n m CM4620 reduced necrosis to a level that was not significantly different from necrosis in control cells (*P *> .05). Therefore, the application of 50 n m CM4620 together with 1 m m galactose had a similar significant effect on POA-evoked necrosis (*P* > .05) to that of 50 n m CM4620 alone. Both 1 n m CM4620 and 10 n m CM4620 alone highly significantly reduced POA-induced cell death rates compared to necrosis due to POA treatment (*P* < .0001, Figure 3B). Addition of galactose significantly decreased necrosis levels as compared to 1 or 10 n m CM4620 alone (*P *< .001 and *P *< .01, respectively, Figure 3B). In fact, a combination of treatments almost entirely inhibited POA-evoked necrosis: CM4620 at all 3 concentrations with galactose decreased necrosis levels induced by POA to the control level (*P *> .05). Galactose alone also significantly protected cells against POA-evoked necrosis (*P* < .0001), similar to our previous results. However, adding 10 or 50 n m CM4620 significantly reduced necrosis further (*P *< .001) as compared to galactose alone. These results demonstrate a synergy between CM4620 and galactose that allows us to use CM4620 at significantly lower concentrations than have been used previously.

While the protective effects of CRAC channel blockers against alcohol or bile-induced PAC injury have been well documented previously,^21^,^33^ their effectiveness in reducing ASNase-elicited toxicity requires further investigation. Therefore, we studied changes in PAC necrosis levels induced by ASNase (200 U/mL) in the presence of 50 n m CM4620, a concentration that has been shown to have a highly significant protective effect in our experiments with POA-induced necrosis (Figure 3B). The results shown in Figure 3C demonstrate that ASNase-elicited necrosis in PACs was very significantly reduced following PAC treatment with 50 n m CM4620 alone (*P *< .0001) and further reduced when used in combination with 1 m m galactose (*P *< .001). Treatment of cells with galactose alone (Figure 3C) decreased ASNase-induced necrosis to a level that is similar to the effect of 50 n m CM4620 treatment. However, using 50 n m CM4620 with 1 m m galactose significantly reduced necrosis (*P *< .001) as compared to galactose alone.

### Effectiveness of Low Concentrations of CM4620 in Combination with Galactose in In Vivo Alcohol Mouse Model of AP

**Figure 4.**
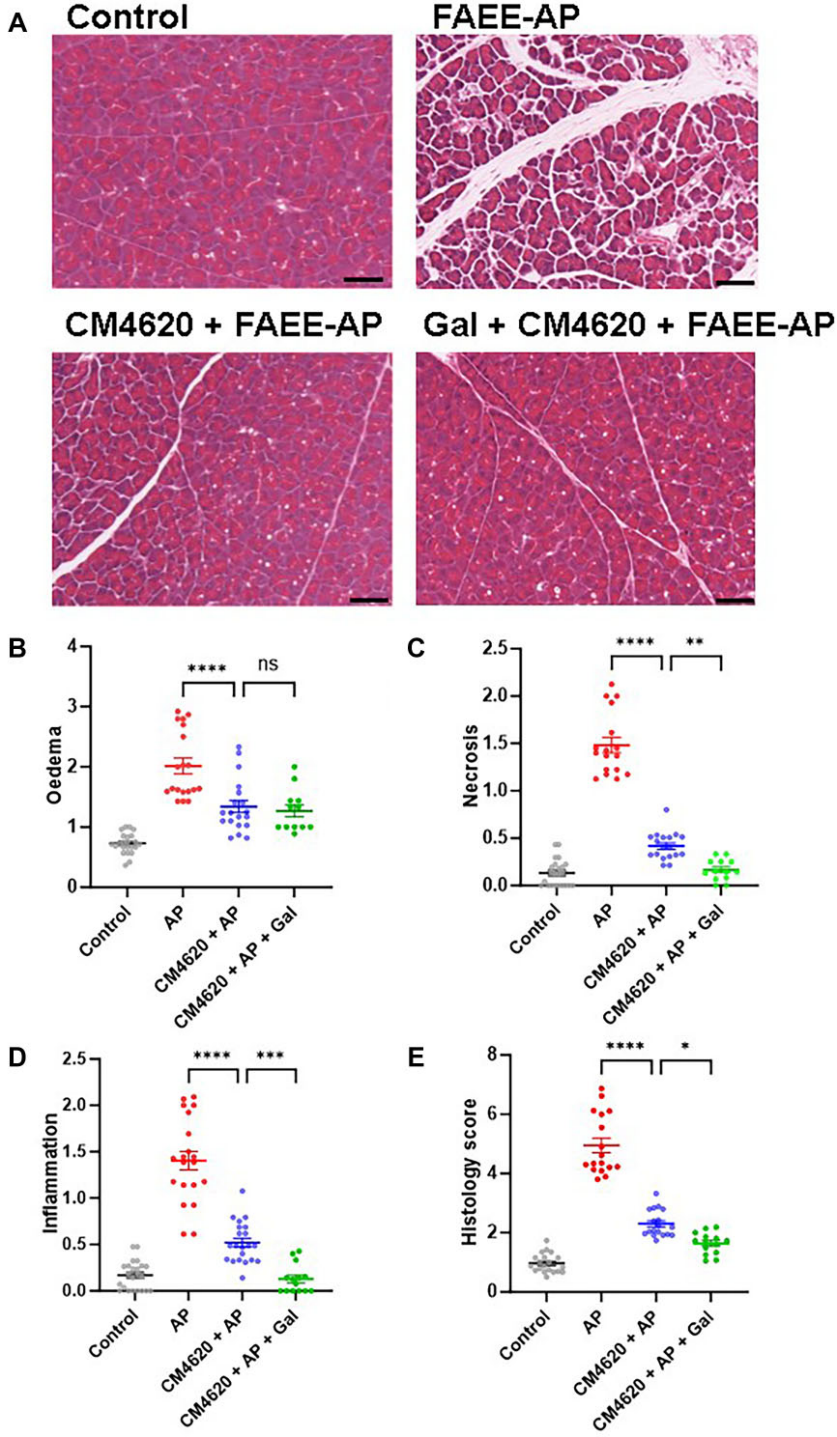
A combination of galactose and a reduced dose of CM4620 markedly diminishes AP development in an *in vivo* experimental mouse model of alcohol-induced AP (FAEE-AP). (A) Representative images of H&E-stained pancreatic acinar tissue sections demonstrate a normal pancreatic tissue histology in control, in FAEE-AP, and in tissue from FAEE-AP mice with CM4620 (0.1 mg/kg) treatment in the absence or presence of galactose (100 m m) supplement in drinking water (CM4620 + FAEE-AP or Gal + CM4620 + FAEE-AP, respectively). Magnification x200, scale bar: 50 μm. (B-E) Significant increases (*P *< .0001) in inflammation (B), necrosis (C), edema (D), and total histology score (E) have been observed in pancreatic tissue of mice with FAEE-AP as compared to control. Administration of 0.1 mg/kg CM4620 via intraperitoneal injections markedly protected against all pathological changes evoked by POA and ethanol (FAEE-AP) *in vivo* (*P* < .0001). Combinational treatment of mice by addition of 100 m m galactose to the drinking water before and during the induction of FAEE-AP with CM4620 injection reduced further necrosis and inflammation (*P *< .01 and *P *< .001, respectively) as well as histological score (*P *< .05). Both necrosis and inflammation have been reduced to untreated control levels (*P *> .05) by the combination of CM4620 and galactose. Experimental groups consisted of ≥4 mice/group. Data are shown as mean ± SEM.

We have now investigated the protective effect of CM4620 at a much lower dose (0.1 mg/kg) than has been previously published^25^,^33^ in an *in vivo* experimental mouse model of AP induced by a mixture of alcohol and POA (FAEE-AP) as described previously. Results shown in Figure 4A-E demonstrate that the injection of 0.1 mg/kg CM4620 alone at the time of FAEE-AP induction significantly reduced the total histological scores (*P* < .0001), including edema (*P* < .0001), necrosis (*P* < .0001), and inflammatory cell infiltration (*P* < .0001), in pancreatic tissue as compared to a positive control (FAEE-AP). A combination of CM4620 with pretreatment of mice with 100 m m galactose in the drinking water significantly improved the histological score (*P *< .05), necrosis (*P *< .01), and inflammation (*P* < .001), whereas it did not significantly improve pancreatic tissue edema (*P *> .05) as compared to CM4620 treatment alone. A combination of treatments also reduced both necrosis and inflammation to control levels (*P* > .05 as compared to untreated control). We believe that such combinational therapy is the way forward in developing potential treatments for AP.

## Discussion

It has been accepted that AP is initiated by intracellular Ca^2+^ overload, causing mitochondrial malfunction, ATP loss, and premature intracellular activation of digestive enzymes, leading to necrosis.^1^,^2^

In physiological Ca^2+^ signaling in PACs, the classical secretagogue ACh and the hormone cholecystokinin (CCK) evoke repetitive cytosolic Ca^2+^ oscillations at physiologically relevant concentrations.^1^,^50^ These oscillations are required for the normal physiological functioning of PACs, namely, the secretion of digestive enzymes and fluid. However, our data show that micromolar concentrations of CM4620 inhibited physiological calcium spikes induced by ACh in PACs (Figure 2A), prompting a need for the reduction of the CM4620 concentration in the treatment of pancreatitis. Our data (Figure 2A-C) show that concentrations of CM4620 at least 10 times lower (<100 n m) are capable of preserving the physiological function of PACs. Although 100 n m of CM4620 displayed only partial inhibition of the calcium entry in PACs, the effect was highly significant (Figure 1), allowing the use of substantially lower concentrations of CM4620.

Recent research for potential AP treatments has been largely focussed on reducing the enhanced Ca^2+^ entry through Orai1/CRAC channels in PACs activated as a result of excessive Ca^2+^ release from internal stores.^1^,^5^,^21^,^33^,^34^,^42^ We have demonstrated previously that prevention of alcohol metabolite–evoked excessive Ca^2+^ entry by blockage of Orai1/CRAC channels with GSK-7975A (GlaxoSmithKline) *in vitro* is beneficial for cell survival.^21^ Our findings have been strengthened later by *in vivo* studies that have demonstrated the protective effect of GSK-7975A and a novel potent Orai1/CRAC channel blocker CM4620 (zegocractin, CalciMedica) against toxicity in AP.^33^,^42^ As a result, the nanoemulsion formulation of CM4620, Auxora, is currently used in several clinical trials in patients with severe AP and COVID-19–induced pneumonia.^39^,^40^,^51^,^52^ It has been demonstrated that Auxora is rapidly distributed to the pancreas and lungs, providing effective inhibition of Orai1/CRAC channels in these tissues.^39^ Recent results from the current Phase 2b, which is a randomized, double-blind, placebo-controlled dose-ranging clinical trial (NCT04681066) of Auxora in patients with AP and associated systemic inflammatory response syndrome and hypoxemia (CARPO)^40^, reported a favorable safety profile of the drug with a significant reduction in the proinflammatory cytokines and the disease severity.^39^,^40^ Previously, it has been shown that the efficacy of CM4620 in preventing histopathological changes of the mouse pancreas in experimental models of AP was potent but incomplete.^33^ Moreover, higher doses or long-term application of this compound could be challenging due to unwanted immunological, muscular, or intestinal side effects.^32^,^41^,^42^ The function of immune cells, as nonexcitable cells similar to pancreatic acinar cells, relies on Ca^2+^ entry mechanisms that involve Orai1/CRAC channels. Therefore, the blockage of these channels by CM4620 would result in a profound inhibition of immune cell response affecting the patients’ recovery after AP and other inflammatory conditions. Previous studies have also observed severe bacterial dysbiosis and the reduction in antimicrobial secretion in Orai1 KO mice within the first 3 wk resulting in up to 70% mortality.^41^ Furthermore, loss-of-function mutations of Orai1 were linked to the increased risk of immunological and muscle disease in humans.^32^ However, current clinical trials of CM4620 for the treatment of severe AP and COVID-19 pneumonia report promising results demonstrating the effectiveness and safety of the drug for patients.^39^,^40^ At present, experimental and clinical data have demonstrated that targeting SOCE by CM4620 is an effective and promising therapeutic avenue for combatting AP.

Recently another Orai1/CRAC channel inhibitor, CM5480, was also successfully used to inhibit the progression of recurrent AP to chronic pancreatitis (CP) by protecting against Ca^2+^ overload in pancreatic acinar and duct cells.^53^,^54^ However, it is also paramount to consider the risks and the benefits of prolonged treatments with Orai1/CRAC channel blockers for patients with AP or CP. It has been demonstrated previously that genetic deletion of Orai1 in PACs in mice caused bacterial outgrowth, dysbiosis, systemic inflammation, and significant mortality.^41^ At the same time, a recent paper suggested that a partial (70%) knockout of Orai1 in the pancreas has protected it against AP but failed to protect against associated lung injury.^61^ On the other hand, specific knockout of Orai1 in neutrophils protected against lung injury but failed to protect against pancreatic damage in AP.^61^ Extension of this work and, in particular, a combination of approaches could provide better results.^62^

In humans, CRAC channelopathies with loss-of-function mutations in Orai1 predispose to severe immunodeficiencies, autoimmunity, muscular hypotonia, and other abnormalities.^32^ Waldron et al. have demonstrated the profound effect of CM4620 on the immune cells in the pancreas.^42^ Therefore, the long-term inhibition of Orai1/CRAC channels with Auxora should be considered with caution. One way to reduce such risks is to reduce the inhibitor concentration to the effective minimum. We have managed to substantially reduce the required CM4620 concentrations to inhibit pancreatic pathology *in vitro*. The lowest effective concentration of CM4620 that significantly inhibited PAC necrosis induced by the POA (30 µm) was 1 n m (Figure 1B). Higher levels of CM4620 were required to significantly decrease levels of cell necrosis induced by bile acids (Figure 3A). Nevertheless, it seems that 50 n m of CM4620 was highly effective for all 3 pathological stimulations *in vitro* (Figure 3A-C). Therefore, we suggest that it is possible to use much lower doses of the inhibitor for effective protection against toxicity induced by the main AP-inducing agents.

Our *in vitro* findings have been used to investigate the potential protective effects of a reduced dose of CM4620 in an experimental *in vivo* mouse model of alcohol-induced AP (FAEE-AP) (Figure 4A-E). We injected mice with 0.1 mg/kg CM4620, which is 20 times lower than the doses reported previously.^33^,^42^ While we did not measure levels of the drug in the pancreas, linear dose dependence was demonstrated in a previous study with CM4620 in mice, allowing us to extrapolate estimated levels of this compound in the pancreas after a single IP dose of 0.1 mg/kg of around 70 ng/mL, which is still above the lower limit of detection of the bioanalytical assay (10 ng/mL) (CalciMedica, unpublished). In addition, because the formulation of the compound in our study used DMSO and PBS, we would expect greater absorption and, therefore, higher concentrations within the tissue than in the previous experiment, so the pancreatic concentration could be higher than 70 ng/mL. Our results demonstrate that treatment with 0.1 mg/kg of CM4620 significantly improved pathological histology scores relevant to AP such as pancreatic tissue edema, necrosis, and inflammation, demonstrating the remarkable potency of CM4620 (Figure 4A-E).

Lower concentrations of CM4620 inevitably reduced the protective effects against AP both *in vitro* (Figure 3A-C) and *in vivo* (Figure 4A-E) as compared to results in earlier reports.^33^,^42^ Nevertheless, our data show that even a relatively small reduction of Ca^2+^ influx into acinar cells was already beneficial for cell survival in AP. However, reduced ATP production remains to be central in the development of the disease. Therefore, boosting ATP production and cellular metabolism with the help of energy supplements such as galactose has synergistically supported cytoplasmic Ca^2+^ clearance by the plasma membrane Ca^2+^ ATPase the plasma membrane Ca^2+^ ATPase (PMCA) and SERCA pumps while restoring Ca^2+^ handling mechanisms and preventing necrotic cell death.^1^,^25^ Therefore, we have considered using a combination of CM4620 with another proposed AP treatment.

We have previously shown that the application of galactose to stimulate ATP production by mitochondria has a significant protective effect against AP *in vitro* and *in vivo*.^25^ Therefore, we have used the addition of 1 m m galactose with low concentrations of CM4620 and found a significant improvement in PAC survival challenged by BA, POA, or ASNase, bringing the degree of necrosis to the control level (untreated cell death rate) (Figure 3C). These results suggest that simultaneous restriction of Ca^2+^ entry by CM4620 and stimulation of ATP production by the energy supplement galactose can allow full compensation for the reduced effect of a low dose of CM4620 for PAC survival. Interestingly, galactose alone also showed a significant reduction of cell necrosis induced by BA, POA, or ASNase. However, a combination with low doses of CM4620 synergistically increased the protective effect of galactose, emphasizing the major role of ATP in PAC survival under pathological conditions.

Similarly to the *in vitro* results, we have shown that the combination of CM4620 with galactose synergistically reduced both necrosis and inflammation parameters to the control levels (Figure 4C and D), as well as significantly reduced the histology score, in an *in vivo* experimental model of alcohol-induced AP in mice. This comprehensive *in vivo* evaluation confirmed the advantage of using Orai1/CRAC channel inhibition and an energy supplement as a novel therapeutic strategy.

Energy supplements^55^ and stimulation of intracellular energy production^56^ have been suggested previously as a treatment for AP. High energy administration in the early phase of AP is being tested in a multicenter, randomized, double-blind clinical trial.^55^ Our recent findings^25^ suggest using the carbohydrate galactose to compensate for ATP in AP. Galactose has a clear advantage for use in clinical studies since it is a natural freely available substance and part of some food products (lactose-free dairy), including baby milk mixtures.^46^ Galactose can be administered by IV injection of up to 0.5 g/kg^57^ or by feeding (drink) protocols.^58^ Clinical trials have shown that oral galactose at a dose of up to 50 g/d can be safely consumed and well tolerated by patients,^47^,^48^ except in very rare cases of galactosemia.^63^ Our new results suggest a need for clinical trials with galactose in combination with low submicromolar doses of CM4620 for patients in the early phase of AP. Such a combination would allow synergistic inhibition of calcium overload, using two independent mechanisms, while reducing the potential unwanted effects of the Orai1/CRAC channel inhibitor. Since galactose works synergistically with the Orai1/CRAC channel inhibitor, it could also help in combination with other inhibitors of Ca^2+^ entry or Ca^2+^ release to reduce Ca^2+^ overload in other pathologies.

## Data Availability

The data underlying this article will be shared on reasonable request to the corresponding author.
